# Towards Regenerative Audiology: Immune Modulation of Adipose-Derived Mesenchymal Cells Preconditioned with Citric Acid-Coated Antioxidant-Functionalized Magnetic Nanoparticles

**DOI:** 10.3390/medicina59030587

**Published:** 2023-03-16

**Authors:** Adeline Josephine Cumpata, Dragos Peptanariu, Ana-Lacramioara Lungoci, Luminita Labusca, Mariana Pinteala, Luminita Radulescu

**Affiliations:** 1 Doctoral School, “Grigore T. Popa” University of Medicine and Pharmacy, Universitatii Street 16, 700115 Iasi, Romania; adeline-josephine_d_cumpata@d.umfiasi.ro (A.J.C.); luminita.radulescu@umfiasi.ro (L.R.); 2 Centre of Advanced Research in Bionanoconjugates and Biopolymers ‘‘Petru Poni’’, Institute of Macromolecular Chemistry Aleea Grigore Ghica, Voda 41A, 700487 Iasi, Romania; peptanariu.dragos@icmpp.ro (D.P.);; 3 Orthopedics and Traumatology Clinic, Emergency Hospital Saint Spiridon, 1 St Independentei Boulevard, 700111 Iasi, Romania; 4 National Institute of Research and Development in Technical Physics Iasi Romania, 700111 Iasi, Romania; 5 ENT Clinic Department, “Grigore T. Popa” University of Medicine and Pharmacy, Universitatii Street 16, 700115 Iasi, Romania

**Keywords:** hearing loss, adipose-derived mesenchymal cells, conditioned media, magnetic nanoparticles, protocatechuic acid, regenerative medicine

## Abstract

*Introduction and Background*: Based on stem cells, bioactive molecules and supportive structures, regenerative medicine (RM) is promising for its potential impact on field of hearing loss by offering innovative solutions for hair cell rescue. Nanotechnology has recently been regarded as a powerful tool for accelerating the efficiency of RM therapeutic solutions. Adipose-derived mesenchymal cells (ADSCs) have already been tested in clinical trials for their regenerative and immunomodulatory potential in various medical fields; however, the advancement to bedside treatment has proven to be tedious. Innovative solutions are expected to circumvent regulatory and manufacturing issues related to living cell-based therapies. The objectives of the study were to test if human primary ADSCs preconditioned with magnetic nanoparticles coated with citric acid and functionalized with antioxidant protocatechuic acid (MNP-CA-PCA) retain their phenotypic features and if conditioned media elicit immune responses in vitro. MNP-CA-PCA was synthesized and characterized regarding size, colloidal stability as well as antioxidant release profile. Human primary ADSCs preconditioned with MNP-CA-PCA were tested for viability, surface marker expression and mesenchymal lineage differentiation potential. Conditioned media (CM) from ADSCs treated with MNP-CA-PCA were tested for Il-6 and IL-8 cytokine release using ELISA and inhibition of lectin-stimulated peripheral blood monocyte proliferation. *Results*: MNP-CA-PCA-preconditioned ADSCs display good viability and retain their specific mesenchymal stem cell phenotype. CM from ADSCs conditioned with MNP-CA-PCA do not display increased inflammatory cytokine release and do not induce proliferation of allergen-stimulated allogeneic peripheral blood monocytes in vitro. *Conclusions*: While further in vitro and in vivo tests are needed to validate these findings, the present results indicated that CM from ADSCs preconditioned with MNP-CA-PCA could be developed as possible cell-free therapies for rescuing auditory hair cells.

## 1. Introduction

Regenerative medicine (RM) involves the use of cells, bioactive molecules and supportive structures for replacing or substituting dysfunctional, lost organs or bodily functions [1]. With more than 20 years of basic and translational research and with the rapidly increasing number of clinical trials testing various regenerative strategies, RM is coming to age, already delivering therapies for so-far intractable diseases [2]. Recent developments in stem cell technology and nanomedicine have offered new perspectives introducing the possibility of rescue and regeneration of musculoskeletal tissues [3], sensory organs [4] and auditory epithelia [5] and to prevent or treat deafness of various causes in the adult and pediatric populations [6]. Differentiation methods to obtain hair cell-like cells seem to be effective; however, they proved to be technically challenging, costly and less amenable to large-scale manufacturing [7]. Mesenchymal stem cells (MSCs) are adult tissues which derive from a large variety of tissues of mesenchymal origin (such as bone marrow, adipose tissue, bone and synovium). MSCs assist regeneration mainly by means of the trophic and immunomodulatory effect generated by their paracrine activity. Adipose-derived mesenchymal cells (ADSCs) are MSCs derived from adipose tissue considered to be a convenient source for RM strategies due to their large-scale availability and phenotypic properties [8]. ADSCs were shown to express immunomodulatory and trophic effects in vitro as well as in vivo in an animal model investigating autoimmune and traumatic hearing loss [9]. Many issues in MSC/ADSC-based cell therapies remain unsolved, especially regarding cell survival after transplantation, accumulation within the target tissue as well as fate surveillance. The currently available clinically approved imaging methods are not able to detect a therapeutic cell population after implantation. Cell fate after implantation cannot be resolved non-invasively, which remains a problem that challenges medium- and long-term follow up of stem cell-based therapy and/or tissue-engineered constructs.

The use of nano-scaled materials, particularly of magnetic nanoparticles (MNPs), has evolved as an increasing field of research and application for medicine and life sciences [10]. Iron oxide MNPs internalized by the cells enable cell maneuverability, making them remotely controllable under an applied magnetic field (MF) as well as being traceable in vivo using clinically available magnetic resonance imaging (MRI). MNPs’ biocompatibility is reportedly excellent, as they can be degraded by already-existent cellular iron handling molecular pathways. MSCs that have incorporated MNPs were shown to retain their specific phenotype and to become remotely controllable using an applied external magnetic field [11] with large applicability for cell targeting and cellular imaging using MRI or magnetic particle imaging (MPI) [12]. ADSCs loaded with MNPs are shown to retain their main phenotypic features in terms of proliferative and differentiation capability [13]. Little is known, however, about the immunomodulatory potential of ADSCs preconditioned with MNPs. MNPs’ interaction with living matter is known to be determined by a their constituent physical parameters such as size, shape and coating materials but also by cell phenotype and functions. As a consequence, every MNP variety and cell type interaction needs to be carefully characterized before considering them as potential therapeutic agents. Several reports indicate that antioxidant drugs are capable of increasing the anti-inflammatory and regenerative potential of ADSCs both in vitro and in vivo [14,15]. To date, MNPs’ mediation of the delivery of antioxidant compounds to stem cells is underexplored but has important potential in increasing the therapeutic efficiency of ADSCs. PCA is a compound found in some traditional Chinese herbs which was reported to exert good antioxidant properties [16]. Citric acid (CA) is an organic compound found in lots of fruits and vegetables and is also known for its antioxidant and regenerative capabilities [17]. In this study, we tested the interaction between iron oxide MNP coated with citric acid (CA) and functionalized with antioxidant-protocatechuic acid (PCA) and human primary ADSCs. The purpose was to detect if ADSCs retain their basic phenotypic features (viability, differentiation, surface markers) when exposed to MNP-CA-PCA as well as to test if conditioned media from MNP-CA-PCA-preconditioned cells elicit an immune response in vitro. The cell viability and retention of characteristic mesenchymal stem cell phenotype of ADSCs exposed to MNP-AC-PCA complexes were tested as well as the in vitro effect of MNP-AC-PCA preconditioning on ADSC cytokine release and interaction with the cellular immune system.

## 2. Materials and Methods

General information

Ferric chloride (FeCl_3_ × 6H_2_O), ferrous chloride (FeCl_2_ × 4H_2_O), 25% ammonium solution, citric acid (CA) and protocatechuic acid (PCA) were purchased from Sigma-Aldrich (USA). The Minimum Essential Medium composed of a mixture of Alpha Eagle 1% Penicillin, Streptomycin and Amphotericin B (10K/10K/25 µg in 100 mL), OsteoImage™ Mineralization Assay and AdipoRed™ Assay Reagent were from Lonza (Verviers, Belgium); the fetal bovine serum (FBS), Tryple, StemPro™ Adipogenesis Differentiation Kit and StemPro™ Osteogenesis Differentiation Kit were from Gibco (Langley, VA, USA); the phosphate-buffered saline (PBS) and Live/DeadTM Cell Imaging Kit were from Invitrogen (Eugene, OR, USA); the CellTiter-Glo 2.0 Assay was from Promega (Madison, WI, USA); the Max DiscoveryELISA kits for human IL6 and IL8 were from Bioo Scientific (Austin, TX, USA) and the Pancoll was from Pan-Biotech (Aidenbach, Germany).

Mouse anti-human antibodies for flowcytometry: anti-CD14-PACIFIC BLUE, anti-CD19-ECD, anti-CD34-PC5, anti-CD90 (Thy-1)-APC-AlexaFluor 750 and anti-CD105-PC7 were purchased from Beckman Coulter (Marseille, France) and Alexa Fluor^®^ 488 Anti-CD73 from Abcam.

Magnetic nanoparticle synthesis

MNP-CA was prepared using the pre-addition method as previously described [18]. Briefly, 20 mL each of FeCl_2_ × 4H_2_O and FeCl_3_ × 6H_2_O were mixed in a molar ratio of 1:2, 1 mL citric acid (0.5 g/mL) and pre-added to the ferrous and ferric solution followed by 40 mL of distilled water. After heating at 65 °C, 14 mL NH_4_OH was added drop-wise into the mixture under vigorous mechanical stirring (650 rpm); the final product was centrifuged and washed 3 times with distilled water. Then, 1 mL solution PCA (10 mg/mL) was added to 1 mL MNP-CA (50 mg/mL) and submitted to mechanical stirring for 15 min before purification by magnetic decantation and washing 3 times with distilled water. The entrapment efficiency of PCA in a CA shell was calculated by absorbance reading at 287 nm of the supernatant solution using the following formula: Entrapment efficiency (%) = 100 (total drug-free amount of drug)/(total amount of drug), where free amount of drug and total amount of drug were determined using the calibration curves for PCA at 287 Nm vs. concentration of free PCA

MNP characterization

FT-IR

The Fourier transform infrared (FT-IR) spectra were recorded on a Bruker Vertex 70 FTIR instrument in a 400–4000 cm^−1^ range, in transmission mode, in KBr pellet.

DLS

The hydrodynamic diameter and zeta potential were recorded using a Delsa Nano C Submicron Particle Size Analyzer (Beckman Coulter, Inc., Fullerton, CA, USA) equipped with a laser diode operating at 658 nm.

STEM images

MNP morphology was analyzed in STEM mode with a Verios G4 UC Scanning electron microscope (Thermo Scientific, Brno, Czech Republic) equipped with an energy-dispersive X-ray spectroscopy analyzer (Octane Elect Super SDD detector, Pleasanton, CA, USA). The STEM studies were performed using the STEM 3+ detector (bright-field mode) at an accelerating voltage of 30 kV. For STEM analysis, the samples were dispersed in water and ultrasonicated, then placed on carbon-coated copper grids with 300 mesh sizes and dried in an oven until the solvent was removed.

Release of antioxidant agent

The release profile of PCA from the MNP-CA-PCA sample was studied in PBS with a pH of 7.4. First, 30 mg loaded magnetic nanoparticles were placed in a 12 kDa dialysis bag and introduced in 100 mL PBS at 37 °C, under gentle stirring. Then, 1 mL of supernatant was taken out at fixed intervals (30 min) and replaced with 1 mL fresh buffer. This 1 mL of supernatant was diluted with 1 mL PBS and then assayed by UV-VIS spectrophotometry at 250 nm. The concentration values of the released PCA were determined using the calibration curve of PCA and the following formula:

C_f’ = Cf + v/V ∑C_(f_(i − 1));

where: v = volume of the release media taken out every time; V = volume measured by UV-VIS; Cf’ = concentration of the released drug and Cf = concentration in volume V at specific intervals.

Free radical scavenging activity by DPPH method

The DPPH method was used for measuring the antioxidant activity of functionalized magnetic nanoparticles. First, 3 mL ethanol solution of DPPH (0.1 mg/mL) was added in each 3 mL suspension of MNP-CA-PCA of different concentrations. After 30 min, the absorbance values were measured using 1 cm quartz cuvettes. The absorbance values were read at 517 nm and the radical scavenging activity was determined using the following equation:

% of inhibition = (Ac − As)/Ac∙100

where: As is the absorbance of MNP-CA-PCA samples of different concentrations and Ac is the absorbance of the DPPH solution of 0.05 mg/mL.

Human primary adipose-derived mesenchymal cells (ADSC)

ADSCs were obtained from healthy donors undergoing liposuction procedures for cosmetic reasons after institutional board ethical approval and informed patient consent was obtained; transportation to the laboratory was carried out in sterile conditions. The resulting lipoaspirate was processed within 24 h as previously described [13]. The lipoaspirate was washed three times with PBS, digested with collagenase type I (0.01 mg/mL) for 2 h at 37.5 °C and centrifuged twice at 300 g for 5 min at RT. The supernatant consisting of tissue debris was removed and the remaining medium further centrifuged at 300 g for 5 min. Pelleted cells were re-suspended in complete culture media (CCM-αMEM with 10% fetal bovine serum and a 1% Penicillin–Streptomycin–Amphotericin B mixture) and counted. Cells were plated at 1 × 106 cells/cm^2^ in appropriate tissue culture flasks (CellBIND surface, Corning). Cells in passage 3–4 were used for experiments, counted automatically and incubated at 37 °C and at 5% CO_2_ in an incubator with the media replaced every 3 to 4 days.

Flow cytometry

For the flow cytometry experiment, ADSCs were cultured in a T25 flask, harvested by detachment with Tryple, washed with PBS twice and finally resuspended in microcentrifuge tubes in 300 μL PBS each, for the unlabeled and labeled samples. The following markers were tested for presence/absence: negative markers for stem cells CD14, CD 19 and CD 34 on the fluorescence channels PB450, ECD and PC5.5 respectively; positive markers for stem cells CD73, CD90 and CD105 on fluorescence channels FITC, APCA750 and PC7, respectively; 1 µL of each antibody was added. Samples were vortexed briefly and incubated for 15 min at 37 °C, centrifuged at 300 g and washed twice with PBS, resuspended in 300 μL and analyzed on a CytoFLEX benchtop flow cytometer (Beckman Coulter Life Sciences, Indiannapolis, IN, USA).

In vitro toxicity of MNPs

In vitro MNP cytotoxicity was tested using the CellTiter-Glo kit. Cells were plated on 96-well white opaque tissue-culture-treated plates at densities of 5 × 103 cells/well in 100 µL/well complete medium and incubated for 24 h. The next day the media were replaced with serial dilutions of magnetite concentrations in complete cell culture medium and the plates were incubated for another 48 h. Before reading the results, the plates were removed from the incubator and kept at RT for 30 min followed by the addition of 100 µL/well of CelltTiter-Glo. Plates were shaken for 2 min and incubated for 15 min at RT. Light emission was assessed by spectrophotometry using the FLUOstar^®^Omega plate reader (BMG, Offenberg Germany). The relative cell viability is expressed as a percentage of the viability of control (cells treated only with cell culture medium) according to the following formula:

relative cell viability = (RLup − RLub)/(RLuc − RLub) × 100

where RLup, RLub and RLuc have relative light units recorded for samples, blank and control wells, respectively.

Live/dead viability assay

ADSCs were plated on 12-well tissue-culture-treated plates at densities of 40 × 103 cells/well in 1 mL/well complete medium and incubated for 24 h. The next day, the media were replaced with coated and non-coated MNPs at a concentration of 63 and 125 μm/mL, respectively; plates were incubated for another 7 days in CCM. At the end of the experiment, component A and component B from the kit were mixed as per the manufacturer’s instructions; plates were incubated at RT for 20 min after which the images were collected using a Leica DMI 3000B inverted microscope (Wetzlar, Germany) using GFP and Texas Red filter cubes.

ADSC differentiation: adipogenesis and osteogenesis

Cells were cultured in 96-well black flat-bottom clear plates for quantitative evaluation and in 12-well plates to be photographed under a microscope. A density of 3200 cells/well was used for the 96-well plates, while 32,000 cells/well were seeded for the 12-well plates in complete αMEM medium. The next day, the medium was replaced with magnetite solutions in αMEM. After another 3 days, the medium was again replaced with commercially available adipocyte differentiation medium, respectively with osteogenic differentiation medium (see general information above). Adipogenesis assay was performed for 11 days while osteogenesis for 19 days as per the manufacturer’s instructions.

Assessment of differentiation: adipogenesis

At 11 days, AdipoRed™ reagent was added according to the manufacturer’s protocol. Briefly, the differentiation medium was removed and the cells were washed with PBS; AdipoRed™ dissolved in PBS was added and the plates were incubated 10 min at RT. Fluorescence (excitation 485 nm; emission 570) was read with a plate reader as described above. Similarly, the plates were qualitatively investigated with fluorescence microscopy.

Assessment of differentiation: osteogenesis

For the osteogenesis assay, cells were fixed with ethanol for 20 min. For qualitative and quantitative evaluation, Osteoimage™ was used as per the manufacturer’s instructions. After staining, the samples were washed 3 times with wash buffer. To quantify the results, the 96-well plates were recorded with the plate reader (excitation 485 nm; emission 505), while the 12-well plates were analyzed with the fluorescence microscope.

Cytokine release

ELISA

ADSCs with or without MNP-AC-PCA were cultured in T25 flasks in αMEM medium with 0.2% FBS and 1% antibiotics for 11 days without changing the medium. After 11 days, the supernatant was removed and kept at −80 °C until the day of the ELISA test. For the ELISA test, steps were followed according to the manufacturer’s protocol. Briefly, 100 µL of 1× assay diluent was added in the negative control wells, 100 µL of interleukin standards in separate wells, as well as 100 µL of the sample in other separate wells and the assay plates were incubated 2 h at RT. After incubation, the liquid was removed, washed 3 times with 250 µL wash solution, 100 µL detection antibody was added to the plates and incubated 1 h at RT, the liquid was aspirated, washed 3 times with 250 µL wash solution, 100 µL 1× avidin-HRP was added and the plates were incubated for 3 h at RT. The wells were washed 3 times with 250 µL wash solution, 100 µL TMB substrate was added and the plates were incubated for 15 min. Then, 100 µL stop buffer was added and absorbance was detected immediately at 450 nm using a plate reader (same as above).

Mixed lymphocyte reaction (MLR)

For MLR, we used the modified protocol by Herzig et al. [19]. First, 5 mL complete blood was collected in a vacutainer containing citrate as anticoagulant. Then, 15 mL Pancoll was placed in a 50 mL tube; 5 mL of blood was mixed with 5 mL PBS and allowed to settle, followed by centrifugation at 900 g for 30 min at 18 °C without the brake. PBMCs were extracted and resuspended, diluted with CCM and counted. The number of replicates for each of the 16 conditions was 3 with 6 × 10 × 5 cells per sample (sample set = 18; there were 16 treated and two controls (one positive and one negative) for lectin (Figure S1, Supplementary Materials). The sample set included conditioned media (CM) from ADSCs with or without MNP-CA-PCA at a 50% concentration (ADSC 50% (50 µL/100 µL) 50 µL concentrated CM and 50 µL medium = 100 µL) and at a 25% concentration (ADSC 25% (25 µL/100 µL) 25 uL concentrated CM and 75 µL medium = 100 µL) in the presence and absence of lectin; 96-well plates were used for this assay. Then, 100 µL per sample was left to incubate for 72 h at 37 °C. PBMC viability/proliferation was determined using 100 μL celltiter-Glo/well; data were processed in GraphPad.

## 3. Results

### 3.1. MNP Characterization 

#### 3.1.1. FTIR

**Figure 1:**
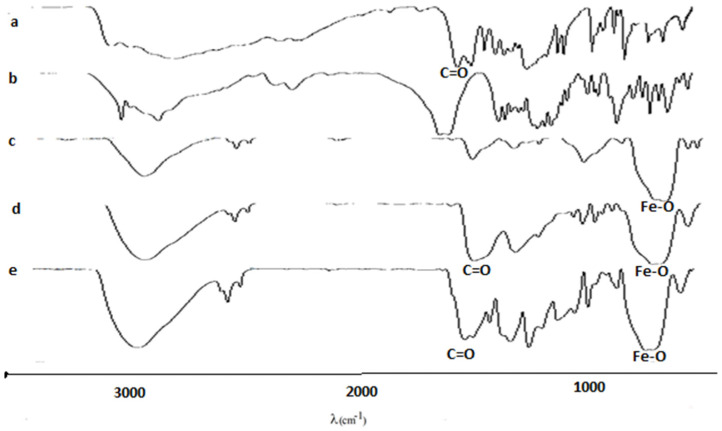
Fourier transformed infrared (FTIR) spectra of magnetic nanocomposites (**a**) protocatechuic acid (**b**) citric acid (**c**) magnetic nanoparticles (**d**) magnetic nanoparticles covered with citric acid (**e**) magnetic nanoparticles covered with citric acid and loaded with protocatechuic acid.

PCA has its characteristic peaks at 1676 cm and 1299 cm (C=O stretching vibration) and at 1467 cm and 1528 cm (C-C aromatic ring stretching mode). Citric acid has its characteristic peaks at 1753 cm (C=O stretch in the carboxylic groups) and in the 1500–1000 domain (C-O, C-OH, C-C vibrations). MNP has a characteristic peak at 572 cm (Fe-O bond). In the spectrum of MNP-CA, we found the characteristic peaks for the Fe-O bond (611 cm) and citric acid (1612 cm C=O stretch). In the spectrum of MNP-CA-PCA we found the characteristic peaks for Fe-O (611 cm), CA (1622 cm) and PCA (1485 cm) (Figure 1).

#### 3.1.2. DLS for the Uncoated MNPs

DLS measurements showed a hydrodynamic diameter of 325.6 nm and a zeta Potential of −2.88 mV. After coating with citric acid, the hydrodynamic diameter increased to 478 nm and zeta potential decreased to −17, 25 mV, which confirms the successful coating with CA. For MNP-CA-PCA, the hydrodynamic diameter was 397.9 nm and the zeta potential was −20.24 mV, which confirmed the adsorption of PCA in the CA shell (Table 1).

#### 3.1.3. STEM

**Figure 2:**
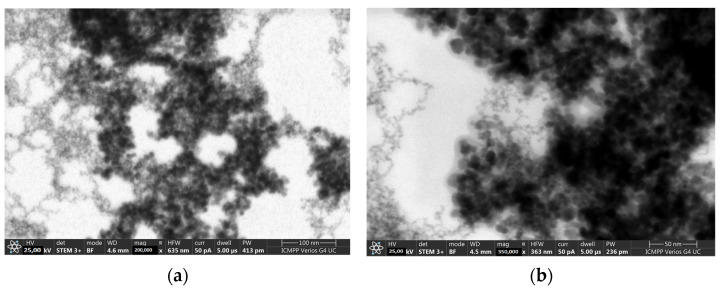
STEM images for MNP (**a**) and MNP-CA (**b**).

The STEM measurements of MNPs showed spherical particles of 7–10 nm with a tendency to agglomerate. For MNP-CA, the STEM images showed the CA coating around the bare MNPs. The difference between DLS measurements and the TEM ones consisted of the fact that in TEM, the solvent evaporated slowly, but in DLS the nanoparticles moved in an aqueous medium, resulting in bigger structures (Figure 2a,b).

#### 3.1.4. EDX 

The EDX spectra of MNPs confirmed the presence of magnetite nanoparticles(Table 2) For MNP-CA and MNP-CA-PCA, the EDX spectra showed the presence of carbon because of the coating with citric acid. For MNP-CA-PCA, the oxygen content was the highest because of the presence of PCA (Table 2).

#### 3.1.5. DPPH 

**Figure 3:**
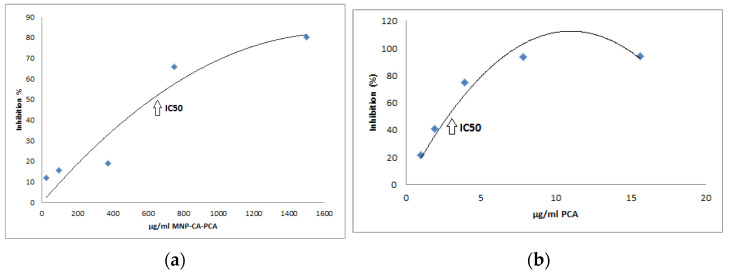
(**a**) MNP-CA-PCA antioxidant activity; (**b**) PCA antioxidant activity.

For MNP-CA-PCA, the DPPH tests indicated an IC50 value of 600 μg/mL. This confirmed good antioxidant activity. Not only did the magnetic core of MNPs not decrease the antioxidant activity of simple PCA but also had an overall contribution in the final antioxidant activity of the MNP-CA-PCA samples (Figure 3a,b).

### 3.2. Drug Release 

**Figure 4:**
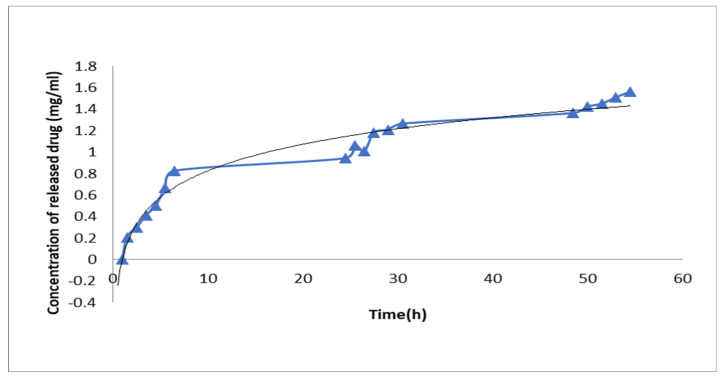

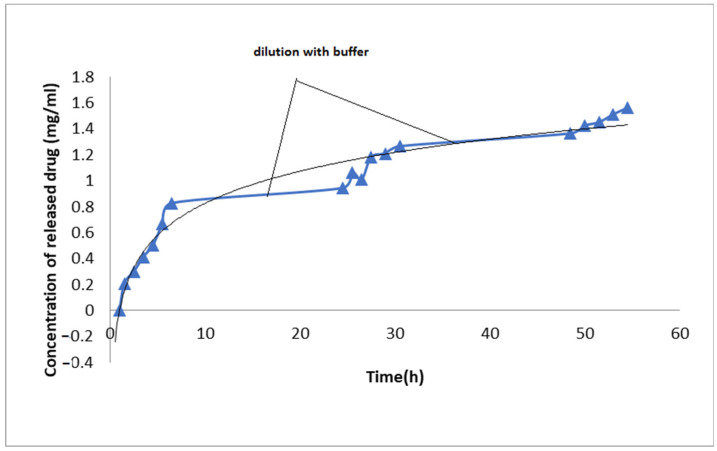
PCA release profile of MNP-CA functionalized with antioxidant PCA. The blue dots represent the number of measurements eash time the release media was taken out ant the straight portion of lines represent the overnight stopping of stirring and temperature over the course of three days.

The drug release profile of MNP-CA loaded with PCA showed a rapid and continuous release of the antioxidant PCA over the course of three days (Figure 4).

#### 3.2.1. In Vitro MNP Cytotoxicity/ADSC Viability

**Figure 5:**
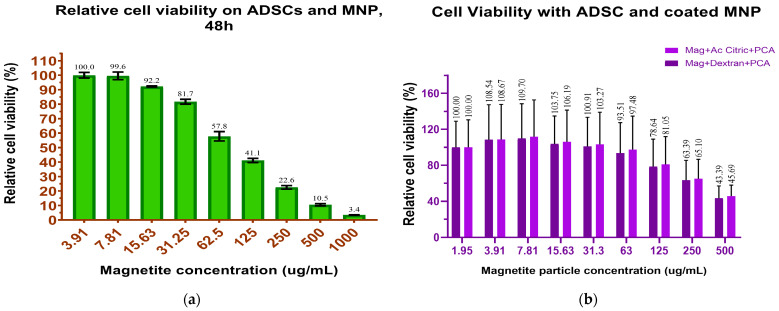
(**a**) ADSC viability with increasing concentrations of as-prepared MNP; (**b**) ADSC viability with MNP coated with dextran and protocatechuic acid D-PCA and citric acid and protocatechuic acid CA-PCA in 63 mg/mL and 125 mg/mL concentrations added in the culture, respectively.

We used two methods to determine cell viability in the presence of MNPs and MNP-CA-PC, respectively. For the quantitative CelltiterGlo test, we firstly tested ADSC viability at 48 h with increasing concentrations of non-coated MNPs in order to determine the particle working concentration. We found that LC 50% was situated between the concentrations of 62.5 and 125 μg/mL (Figure 5a). Next, dextran and citric acid-coated MNPs containing PCA in concentrations of 62.5–125 μg/m were tested for long-term viability against ADSCs. Since MNP-CA-PCA exposure yielded higher viability at 7 days, this coating was chosen to proceed with further experiments (Figure 5b).

**Figure 6:**
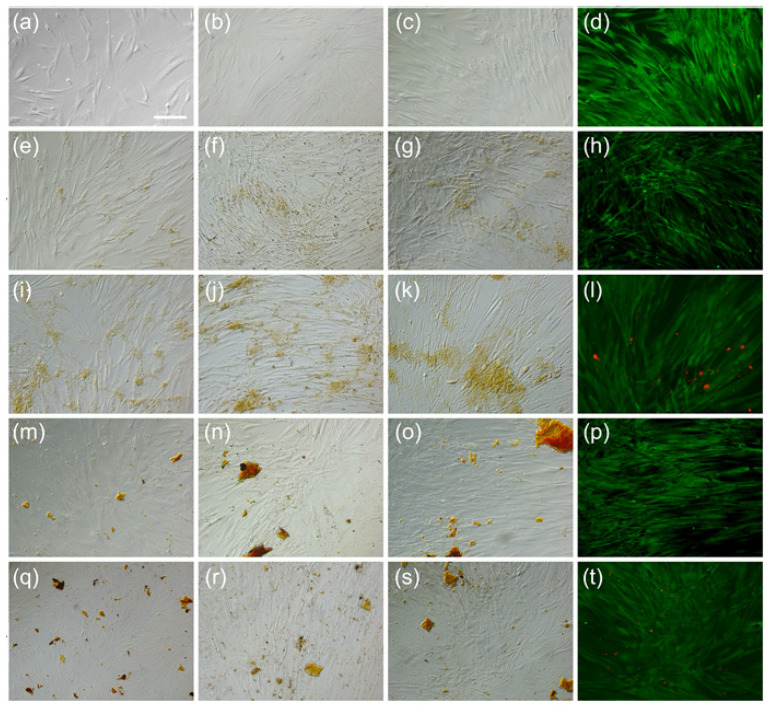
LIVE/DEAD qualitative evaluation of ADSC viability in the presence of MNP. (**a**–**d**) ADSC from day 0 to day 7 (in that order), bright field (BF) d 10×. (**e**) LIVE/DEAD staining at day 7 after LIVE/DEAD fluorescence, 10×. (**f**–**h**) ADSC-MNP-CA-PCA 63 μg/mL at days 3, 5 and 7, respectively BF 10×; (**i**) ADSC-MNP-CA-PCA LIVE/DEAD 7 day fluorescence. (**j**–**l**) ADSC-MNPs of 125 μg/mL at days 3, 5 and 7 BF 10×; (**m**) ADSC-MNP LIVE/DEAD day 7 fluorescence at BF 10×. (**n**–**p**) ADSC-MNP-D-PCA 63 μg/mL, BF 10×; (**q**) ADSC-MNP-D-PCA 7 day LIVE/DEAD fluorescence BF 10×; (**r**,**s**) ADSC-MNP-D-PCA 125 μg/mL, BF 10×; (**t**) ADSC-MNP-D-PCA 125 μg LIVE/DEAD, fluorescence, BF 10×; ADSC = cells non exposed to magnetic nanoparticles; ADSC-MNP = cells exposed to as-prepared magnetic nanoparticles; ADSC-MNP-CA-PCA = ADSC exposed to magnetic nanoparticles coated with citric acid and functionalized with protocatechuic acid; ADSC-MNP-D-PCA = ADSC exposed to magnetic nanoparticles coated with dextran and functionalized with protocatechuic acid.

Qualitatively, ADSC viability was tested using the LIVE/DEAD assay. The method is based on the use of two fluorescent dyes for cell staining: a membrane-permeable dye for living cells and an impermeable dye to mark dead cells. The two dyes can be observed microscopically. The living cells display an intense fluorescence in the green domain (ex/em 488 nm/515 nm), while dead cells emit in the red domain (ex/em 570 nm/602 nm). We found that ADSCs in the presence of MNP-CA-PCA and MNP-D-PCA, 63 and 125 μg/mL, displayed an increased number of dead cells for both types of coatings for 125 μg/mL as compared with 63 μg/mL (Figure 6).

#### 3.2.2. ADSC Differentiation

**Figure 7:**
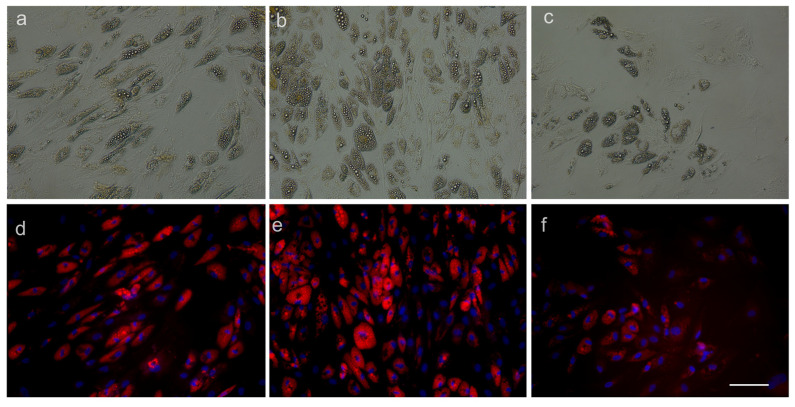
Adipogenic differentiation of ADSCs exposed and not exposed to magnetic nanoparticles: (**a**) ADSC-MNP-CA-PCA 63 μg/mL adipogenic differentiation after 10 days, BF 20×.; (**b**) ADSC-MNP-CA-PCA 125 μg/mL; (**c**) ADSCs not exposed to MNP ((**a**–**c**) bright field); (**d**) ADSC-MNP-CA-PCA 63 μg/mL fluorescence (Adipored). The lipids are reddish-orange and the nuclei are blue (Hoechst); (**e**) ADSC-MNP-CA-PCA 125 μg/mL fluorescence (AdipoRed); (**f**) ADSCs not exposed to MNP (**d**–**f**) fluorescence (Adipored). The scale bar is 100 μm. Representative results are from one donor.

We tested the differentiation potential of ADSCs cultured in specific, commercially available adipogenic and osteogenic differentiation media in the presence and absence of magnetic nanoparticles. The aim was to assess if the presence of MNP interferes with one of the definitory traits of mesenchymal progenitors: mesenchymal lineage differentiation. For the qualitative assessment of adipogenesis, we used AdipoRed^®^. The AdipoRed^®^ reagent is a commercially available fluorescent dye used to color intracellular fat droplets because it is associated with triglycerides and in this way provides an image of the stage of adipocytic differentiation. It is a more sensitive assay than other methods such as the Oil Red O assay. ADSCs exposed and not exposed to as-prepared MNPs, MNPs loaded with citric acid or dextran, respectively and functionalized with PCA displayed positive AdipoRed^®^ staining after 10 days in culture, signifying the successful conversion to preadipocytes. This was true for both concentrations of MNPs at 63 and 125 μg, respectively (Figure 7).

**Figure 8:**
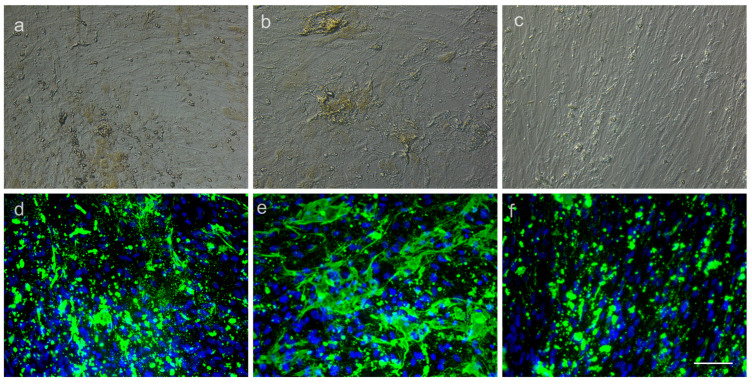
Osteogenic differentiation of ADSCs exposed and not exposed to MNP after 19 days in culture: (**a**) ADSC-MNP-CA-PCA 63 μg/mL osteogenic differentiation after 19 days, BF 20×; (**b**) ADSC-MNP-CA-PCA 125 μg/mL; (**c**) ADSCs not exposed to MNP ((**a**–**c**) bright field); (**d**) ADSC-MNP-CA-PCA 63 μg/mL fluorescence (OsteoImage^®^). The deposited mineralized extracellular matrix is green and the nuclei are blue (Hoechst); (**e**) ADSC-MNP-CA-PCA 125 μg/mL fluorescence OsteoImage^®^ (**f**) ADSCs not exposed to MNP (**d**–**f**) fluorescence OsteoImage^®^ 20×. The scale bar is 100 μm. Representative results are from one donor; ADSC-MNP = cells exposed to non-coated magnetic nanoparticles; ADSC-MNP-CA-PCA = ADSC exposed to magnetic nanoparticles coated with citric acid and functionalized with protocatechuic acid; ADSC-MNP-D-PCA = ADSC exposed to magnetic nanoparticles coated with dextran and functionalized with protocatechuic acid.

For assessing osteogenic conversion, we used fluorescent OsteoImage^®^ dye (Lonza) that specifically binds to mineralized extracellular matrices deposited by pre-osteoblasts and osteoblasts if osteogenic conversion is successful. We found that ADSCs exposed and not exposed to as-prepared MNPs, MNPs loaded with citric acid or dextran, respectively and those functionalized with PCA displayed positive OsteoImage^®^ staining after 19 days in culture, signaling osteogenic conversion. This was true for both concentrations of MNPs, i.e., 63 and 125 μg/mL, respectively (Figure 8).

**Figure 9:**
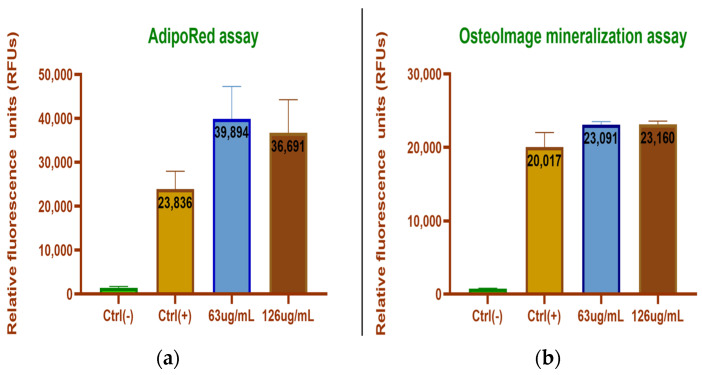
Quantitative differentiation assay. (**a**) Adipogenic differentiation of ADSCs exposed and not exposed to magnetic nanoparticles MNP-CA-PCA; (**b**) osteogenic differentiation of ADSCs exposed and not exposed to MNP-CA-PCA.

Quantitative evaluation of adipogenesis, respectively, osteogenesis using a spectofotometric assesment of fluorescent AdipoRed^®^ and OsteoImage^®^ revealed that MNP-CA-PCA-loaded cells in both concentrations displayed increased differentiation potential compared with non-MNP-loaded controls (Figure 9).

#### 3.2.3. Mesenchymal Stem-Cell-Specific Surface Markers

**Figure 10:**
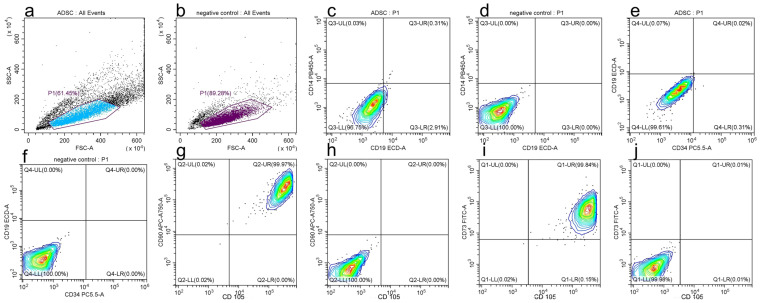
Flow cytometry evaluation of surface markers. (**a**) gating around all significant events, (**b**) gating around the events in the negative control, (**c**) CD 14 and CD 19 where a cluster can be observed in the negative quadrants signifying the absence of CD 14 and CD 19, (**d**) negative control for CD 14 and CD 19, (**e**) Cell cluster CD 19 and CD 34 found in the negative quadrants, (**f**) negative control for CD 19 and CD 34, (**g**) CD 90 and CD 105 cell clusters found in the positive quadrants signifying the presence of these markers in the ADSCs, (**h**) negative control CD 90 and CD 105 in the negative quadrants, (**i**) Cell clusters CD 90 and CD 73 in the positive quadrants and (**j**) negative control CD 73 and CD 105 in the negative quadrants.

Flow cytometry was used to identify positive and negative cell markers that define the phenotype of mesenchymal stem cells and identify the cells tested as being ADSCs. The positive markers for these cells are CD 73, CD 90 and CD 105. The negative markers or the markers that should not be present on ADSCs are CD 14, CD 19 and CD 34. Figure 9 depicts the presence of the positive markers and the absence of the negative markers for ADSCs representative of one donor (Figure 10).

#### 3.2.4. Cytokine Release

**Figure 11:**
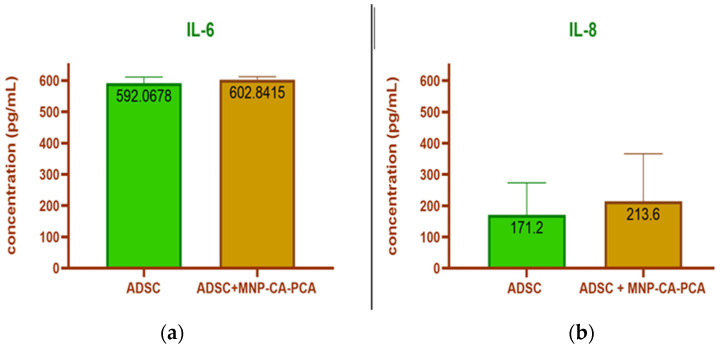
Cytokine release in CM from ADSCs preconditioned with MNP-CA-PCA: (**a**) IL-6; (**b**) IL-8. In green adipose derived stem cells (ADSC) cytokine release in brown adipose derived stem cells treated with MNP-CA-Pca cytokine release.

The alteration of the anti-inflammatory profile of ADSCs in the presence of MNPs was investigated by measuring the level of interleukins IL-6 and IL-8 released in serum-free culture media using the ELISA technique. We found that after 11 days in culture, ADSCs and ADSC-MNP-AC-PCA release had strikingly comparable levels of IL-6, averaging 590/609 pg/m, while non-significant increases of IL-8 could be detected in ADSC-MNP-AC-PCA (averaging 171/2 compared with 213/6 pg/m (Figure 11a,b).

#### 3.2.5. MLR

**Figure 12:**
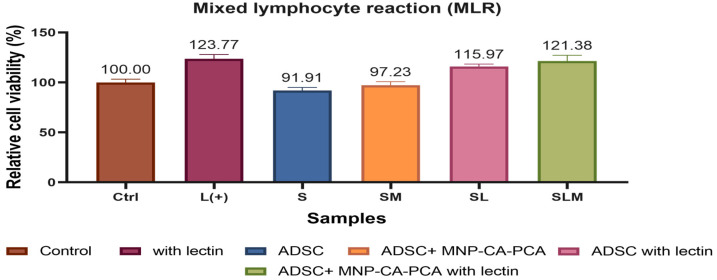
The effect of CM from ADSC-CA-MNP on the proliferative reaction of lectin-stimulated allogeneic peripheral blood monocytes (PBMC) tested using CellTiter-Glo viability assay.

We tested the ability of conditioned media from ADSCs and ADSC-MNP to influence peripheral blood monocyte reactivity to induce stimulation with a common allergen (lectin). As expected, fresh isolated human PBMCs in the presence of lectin displayed higher viability than non-stimulated PBMCs, showing their reactivity and induced cellular immune response. We found that conditioned media from ADSCs inhibited the proliferation of non-stimulated PBMCs by 10%, while conditioned media from ADSC-MNP resulted in inhibition of proliferation by 3%. A similar profile was recorded in the case of lectin-stimulated PBMC-conditioned media from ADSCs, which decreased lectin-stimulated PBMC proliferation by 7.7%, while conditioned media from ADSC-MNP decreased stimulated PBMC only by 0.3% (Figure 3). While ADSCs have the ability to decrease proliferation of allergen-stimulated PBMCs, albeit non-significantly, MNP treatment decreases this ability. Lectin-stimulated as well as naïve PBMCs, however, did not increase proliferations in the presence of ADSC-MNP-conditioned media in this model in vitro (Figure 12).

## 4. Discussion

Recently, the use of nanoparticles and especially MNPs has elicited constant interest for the development of potentially novel breakthrough therapies. MNPs’ relevance as drug delivery systems constitutes a special field of research and their relevance for regenerative medicine is increasingly recognized. Given the significance of immune reactivity in regenerative processes, the design of therapeutic agents that have the ability to modulate soluble and cellular immune responses is of the utmost importance [20]. The use of stem-cell-based therapies as regenerative and immune-modulatory agents has already been tested in clinical trials for a large variety of therapeutic applications [21]. Stem-cell-based regenerative strategies for congenital or acquired hearing loss are expected to offer improved therapies [22]. However, the complexity of producing and marketing products that are based on living cells in terms of manufacturing, costs and ethical and regulatory approval has consistently impaired their advancement to clinical settings. Serum-free conditioned media (CM) from ADSCs has been sought out as a modality to avoid the hurdles involved in the therapeutic delivery of living cells [23]. CM from ADSCs preconditioned with various molecules has proven efficient in rescuing hair cell loss due to paracrine regenerative and immunomodulatory activity [24].

In this study, CM from human primary ADSCs preconditioned with MNPs coated with citric acid and antioxidant protocatechuic acid were tested for their effects on immunomodulatory cytokine release and in limiting cellular responses initiated in allogeneic human PBMC by a known allergen (lectin) using a modified protocol [19].

In-house-synthetized MNP-CA-PCA proved to have regular geometry, excellent dispersibility and good colloidal stability (zeta potential −24, 2 compared with as-prepared MNPs (−2), certifying the method used for coating and functionalization results in MNPs with superior characteristics related to their interaction with living matter.

Herein, we reported the synthesis and characterization of a novel formulation of core-shell iron oxide MNPs coated with CA and functionalized with antioxidant molecule PCA as well as their interaction with ADSCs.

The adsorption of an organic shell on the surface of MNPs allows the stabilization of MNPs, decreasing their tendency to agglomerate. Additionally, the zeta potential of coated MNPs tends to increase (positive or negative), making them more suitable for biomedical applications [25].

Furthermore, the core-shell nanoparticles can be used for loading therapeutic agents (drugs, redox substances) which can release the molecules at the site of interest [26].

We tested cell viability using two distinct methods, i.e., a qualitative (LIVE/DEAD) and a quantitative one (CellTiter-Glo) as a modality to investigate a larger panel of enzymatic equipment that is mandatory for cell viability and/or proliferation. The CellTiter-Glo test is based on the reaction between the luciferase enzyme and its substrate (luciferin), a reaction catalyzed by cellular ATP that results in the formation of oxyluciferin and the emission of photons. Since the efficiency of the reaction depends on the amount of ATP, and the latter depends on the number of living cells, the CellTiter-Glo test indirectly measures cell viability as well as cell proliferation. The CellTiter-Glo test is much more reliable than the colorimetric tests in which the absorbance of the chemicals to be tested can be difficult to subtract from the calculations and where the chemicals can produce reactions with the reagents of the test kits, leading to interference [27]. LIVE/DEAD detects the presence of intact cellular membranes as well as the presence of enzyme esterase activity characteristic of viable cells. Both tests performed indicated the excellent viability of ADSCs in the presence of MNP- CA-PCA added in the culture media and allowed to establishment of a working concentration for further experiments (i.e., 63 and 125 μg/mL, respectively). Previous studies reported good to excellent biocompatibility of MNPs with different coatings interacting with ADSCs in vitro as well as in vivo [28,29]. Good viability was reported in different mesenchymal stem cell types upon internalization of commercially available MNPs with different coatings [30]. Herein, we reported that human primary ADSCs interacting with as-prepared custom-made magnetite (Fe_3_O_4_) MNP as well as MNPs coated with citric acid and antioxidant PCA display excellent short- and medium-term viability up to 7 days in culture. Compared with MNP-D-PCA, we found cell viability to be increased when exposed to MNP-CA-PCA, albeit non-significantly, at every concentration, therefore used we used the latter for further experiments. This effect was found to be, however, dose dependent since cell viability dropped below 50% at 50 μg/mL added in culture media (Figure 3). A particularity of MNPs that governs their interaction with cells and is distinctive from other nanoparticles is the existence of an intrinsic mechanism for iron storage and metabolism. Ferritin-dependent iron metabolism is an endogenous mechanism ubiquitous and evolutionarily conserved in all mammalian cells. Ferritin protein cages were shown to store degradation products of MNP cores, concomitantly slowing down the process due to their colloidal behavior in the acidic medium of the lysosomes [31]. A possible biosynthesis of bio magnetite by human mesenchymal stem cells after MNP degradation has been proposed as well, a process that might be cell-state-dependent [32].

We next tested if human primary cell interaction with MNP-AC-PCA interferes with their basic phenotypic features. The defining characteristics of ADSCs consist of their ability to adhere to a plastic surface of a culture dish, expression of CD73, CD90 and CD105, and lack of CD11b or CD34, CD19 or CD79, CD45 and HLA-DR as well as trilineage mesenchymal differentiation [33,34]. We found that after being exposed to different concentrations of as-prepared MNPs and CA-PCA-coated MNPs, ADSCs retained their adherence to a plastic culture dish for up to 7 days in culture. Cells retained their characteristic surface marker profile (Figure 10) and were capable of undergoing adipogenesis as well as osteogenesis in the presence of MNP-CA-PCA at 63 μg/mL as well as 125 μg/mL (Figure 7, Figure 8 and Figure 9). Moreover both adipogenesis as well as osteogenesis were increased in the presence of MNP-CA-PCA, a fact that suggests cells not only preserve but increase their differentiation potential. Similar reports using commercially available [35] or proprietary MNPs with various coatings [27] indicate that ADSCs retain their differentiation potential to mesenchymal lineages in the presence of MNPs with various coatings. Enhanced cell metabolism produced by the presence or iron as well as changes in cell shape and cytoskeletal rearrangement were proposed to explain this finding. Several groups reported the increased osteogenic potential of bone marrow mesenchymal stem cells (BMSCs) loaded with MNPs [36,37]. Adipogenesis and increased osteogenesis were previously reported in ADSCs incorporating MNPs [27,38], a process that appears to, however, decrease with increasing concentrations of MNPs [39,40]. Herein, we found that both 63.5 as well as 125 μg/mL MNP-CA-PCA support ADSC adipogenesis and osteogenesis in vitro in the experimental conditions created.

We then tested the effect of MNP-CA-PCA presence on inflammatory cytokine expression by ADSCs. Serum-free CM from ADSCs treated with 125 μg/mL MNP-CA-PCA were found to release comparable levels of IL-6 and slightly non-significant increased levels of IL-8. IL-6 is a common denominator of acute inflammation and member of the pro-inflammatory cytokine family [41]. Since the presence of MNPs does not increase its release, it is possible ADSCs do not acquire an inflammatory phenotype in their presence. A slight increase in IL-8, another pro-inflammatory cytokine which can, however, act as an anti-inflammatory myokine [42], can possibly indicate ADSC reactivity. This process is very likely to be donor-dependent since very different values were obtained in CM media from different donors.

MSCs and especially ADSC cell suspension, CM or CM-derived extracellular vesicles are known to inhibit proliferation of allergen-stimulated allogeneic monocytes [43,44]. Herein, we found that CM from ADSCs but also from ADSC-CA-PCA reduce allogeneic blood monocyte proliferation under non-allergen stimulation, which indicates their ability to modulate cellular immune responses in vitro. This is a very important finding that has not been reported before which promotes the use of cell-free regenerative solutions. Hearing loss of various degenerative, traumatic congenital abnormalities [45] could benefit from such therapeutic approaches. Previous reports indicate that free iron ions are potentially capable of promoting immune modulation by stem cells [46] in a process that could potentially be similar to macrophage polarization. This process is very likely fine-tuned with many factors involved (including ferric/ferrous iron balance, transferrin and hepcidin protein activity) [47]; therefore, our findings need to be further tested in vivo. Further tests are warranted to detect the potential of MNP-CA-PCA-preconditioned allogeneic ADSCs both in vitro and in vivo and to discriminate donor-related differences regarding ADSC immune profile. The present findings lay the foundation for and justify further investigation of the use of CM from preconditioned ADSCs as regenerative therapies for rescuing auditory hair cells and for treating hearing loss, with the important potential to translate the aforementioned to clinically available therapies.

## 5. Conclusions

This is, to our knowledge, the first report on the effect of MNP-CA-PCA preconditioning on ADSC phenotype retention and immune profile. We found that ADSC pretreatment with 63.5 and 125 μg/mL did not interfere with stem cell phenotype and supported cells’ immune modulatory activity. Further tests are needed to validate the use of CM from MNP-CA-PCA-preconditioned ADSCs to rescue auditory hair cells and potentially in other sensory epithelia. These findings are important for the design of regenerative approaches to address hearing loss with important potential to translate them to clinically available therapies.

## Figures and Tables

**Table 1:** DLS measurements for MNP, MNP-CA and MNP-CA-PCA.

Sample	Hydrodynamic Diameter (nm)	Zeta Potential (mV)
**MNP**	**325.6**	**−2.88**
**MNP-CA**	**478**	**−17.25**
**MNP-CA-PCA**	**397.9**	**−20.24**
**Title 1**	**Title 2**	**Title 3**
entry 1	data	data
entry 2	data	data

**Table 2:** EDX measurements for MNP, MNP-CA and MNP-CA-PCA.

Sample	Fe%	O%	C%
**MNP**	**84.9**	**15.1**	**0**
**MNP-CA**	**95.8**	**0.3**	**3.9**
**MNP-CA-PCA**	**67.1**	**24.4**	**8.5**

## Data Availability

Data are available from authors at reasonable request.

## Supplementary Materials

The following supporting information can be downloaded at: https://www.mdpi.com/article/10.3390/medicina59030587/s1, Figure S1: MLR experiment set up details.
