# Supplementary material for: Towards Regenerative Audiology: Immune Modulation of Adipose-Derived Mesenchymal Cells Preconditioned with Citric Acid-Coated Antioxidant-Functionalized Magnetic Nanoparticles
Source: Medicina (Kaunas). 2023 Mar 16;59(3):587. doi: 10.3390/medicina59030587 (PMC10058393; doi:10.3390/medicina59030587)
Supplement: Supplementary file 1 [file medicina-59-00587-s001.zip › medicina-2259553-Supplementary material.pdf]

D3 CL + S 50% (3 replicates)	D3 CL + S 25% (3 replicates)
D3 CL - S 50% (3 replicates)	D3 CL - S 25% (3 replicates)
D3 ML + S 50% (3 replicates)	D3 ML + S 25% (3 replicates)
D3 ML - S 50% (3 replicates)	D3 ML - S 25% (3 replicates)

L+, L-

D4 CL + S 50% (3 replicates)	D4 CL + S 25% (3 replicates)
D4 CL - S 50% (3 replicates)	D4 CL - S 25% (3 replicates)
D4 ML + S 50% (3 replicates)	D4 ML + S 25% (3 replicates)
D4 ML - S 50% (3 replicates)	D4 ML - S 25% (3 replicates)

Figure S1 Supplementary material MLR experiment set up and working protocol details Representative data from two donors tested in parralel. D3= donor, D4= donor, CL= control + lectin, +S= ADSC supernatant, -S= no supernatant, ML= magnetite + lectin, L+= lectin, L-= complete medium with no lectin

The materials used for the mixed lymphocyte reaction were: one 50 mL tube, 5 mL complete blood collected in a vacutainer containing citrate, 5 mL plain PBS (without FBS) and 15 mL of Ficoll. The entire quantity of Ficoll (15 mL) was placed in a 50 mL tube after which 5 mL of blood was mixed with 5 mL of PBS and was allowed to settle on top of the Ficoll layer. Afterward, the solution was centrifuged at 900 g for 30 min at 18°C without the brake. The PBMCs were extracted and resuspended. The cells were diluted with complete medium and were counted. The number of replicates for each of the 16 conditions were 3 and 600, 000 cells were needed per condition (sample set= 18; there were 16 conditions and two controls (one positive and one negative) for lectin (Figure S1).

The sample set included the supernatant of ADSCs with MNPs and without MNPs at a 50% concentration (ADSC 50% (50uL/100uL) 50uL concentrated supernatant and 50uL medium= 100uL) and at a 25% concentration (ADSC 25% (25uL/100uL) 25uL concentrated supernatant and 75uL medium = 100uL) for both donors D3 and D4, in the presence and absence of lectin
